# Novel Development of a Large Cerebral Cavernous Malformation in an Adolescent With a History of Familial Cerebral Cavernous Malformation Syndrome

**DOI:** 10.7759/cureus.52591

**Published:** 2024-01-19

**Authors:** Mary G McIntosh, Laura L Hayes

**Affiliations:** 1 Radiology, College of Medicine, University of Central Florida, Orlando, USA; 2 Pediatric Radiology, Nemours Children's Hospital, Pensacola, USA

**Keywords:** neuroradiology, pediatric neuroradiology, vascular anomaly, familial cerebral cavernous malformations, cerebral cavernous malformations

## Abstract

Cerebral cavernous malformations (CCM) are capillary vascular malformations of the central nervous system (CNS). These lesions can be either familial or sporadic. We present a case of a 16-year-old girl with familial CCM syndrome who presented with a six-month history of chronic headaches. A magnetic resonance imaging (MRI) scan revealed a large cavernoma in the right frontal lobe that had not been present on a prior scan performed eight years earlier. This case presentation demonstrates the possibility of significant novel cavernoma development further into adolescence.

## Introduction

Cerebral cavernous malformations (CCM) are structurally anomalous, slow-flow capillaries of the central nervous system (CNS) [1]. They can be sporadic (typically consisting of a single lesion) or familial due to an autosomal dominant mutation (typically associated with multiple lesions) [1]. Magnetic resonance imaging (MRI) is the most sensitive and preferred method of detection, and CCM typically appear popcorn-like and heterogeneous on MRI with a hemosiderin rim [2]. They are most conspicuous on susceptibility-weighted images. CCM often present asymptomatically, particularly the sporadic form, which makes up approximately 80% of cases. They may also present with focal neurologic deficits, seizures, or headaches, often due to hemorrhage [3]. We present a case of familial CCM in which the patient was screened using MRI at the ages of eight and 16 years. In this case, the second scan revealed a new, large right frontal lobe CCM that was not present in the prior scan. Familial CCM has demonstrated a dynamic nature, and this case highlights the possibility of significant new lesion formation occurring at least beyond the age of eight years [4].

## Case presentation

A 16-year-old girl presented with a six-month history of chronic headaches. Non-steroidal anti-inflammatory drugs (NSAIDs), including ibuprofen, had been used prior to her diagnosis and were helpful in the management of headaches. Physical examination and basic lab work were normal.

The patient had an established diagnosis of autosomal dominant familial cerebral cavernoma syndrome. The specific CCM mutation has not been identified because genetic testing has not been performed due to family concerns for possible insurance repercussions. The patient’s sister was evaluated by a geneticist, and after a thorough review of the family history, was diagnosed with an autosomal dominant familial cerebral cavernoma syndrome. The mutation has been passed down from the maternal grandmother’s family that originated in Sweden. The patient’s mother, sister, grandmother, great-grandmother, aunts, uncles, and cousins have been diagnosed with CCM confirmed on MRI scans. Most family members presented with headaches or seizures. 

**Figure 1:**
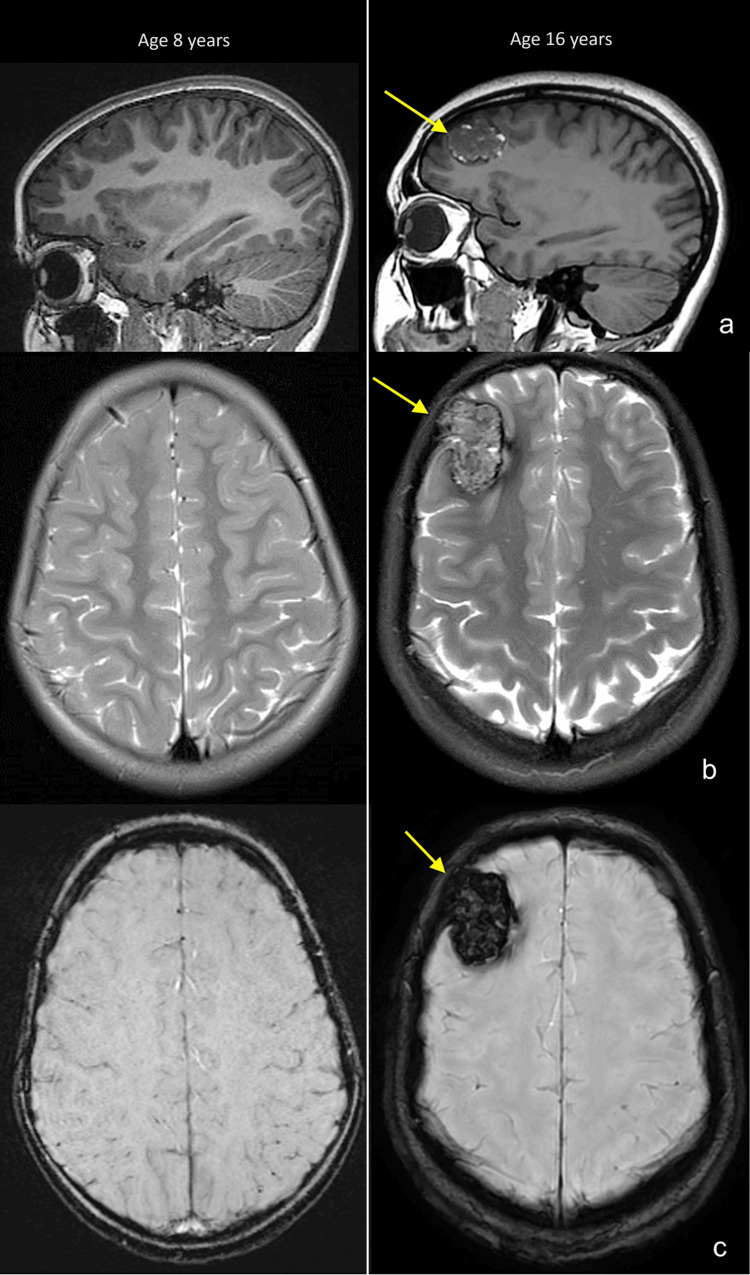
Sagittal T1 (a), axial T2 (b), and axial susceptibility-weighted images (c) from MRI scans of the patient at age eight years (left) and 16 years (right) demonstrate interval development of a new, large right frontal lobe cavernoma (arrows). MRI, magnetic resonance imaging

A headache intractable to NSAIDs, rizatriptan, and lifestyle modifications including increased sleep and water intake and lasting several days ultimately prompted imaging. The MRI scan demonstrated a 4 cm cavernous malformation in the right frontal lobe with minimal surrounding edema and associated endosteal scalloping of the right frontal bone (Figure 1). Only one prior MRI scan had ever been performed due to an established diagnosis when the patient was eight years of age (Figure 1). The right frontal lobe cavernoma had not been present on the prior MRI scan of the brain eight years prior. The scan at age eight years was normal except for one tiny focus of blooming in the pons on the susceptibility-weighted sequence that remained stable on the follow-up scan. The first scan at age eight years was performed with the Siemens Trio 3T scanner, and the second scan at age 16 years was performed with the GE Signa Architect 3T scanner. An electroencephalogram (EEG) was done at age 16 years and was normal.

Given the abnormal scan, strong family history of seizures in the setting of CCM, and high false-negative rate of routine EEG, an anticonvulsant was given as a precaution. A 48-hour EEG was performed, but the results were not available at the time of this report. The patient was managed with acetaminophen as needed for headaches. The patient was cautioned to avoid medications with anticoagulant properties, such as ibuprofen, and herbal remedies, such as Feverfew (Now Foods, Bloomingdale, IL). She has done well on this management regimen and has not required surgical intervention. As the scan was performed less than one year ago and the patient is doing well, no further scans have been performed.

## Discussion

Studies are lacking regarding the natural history of familial CCM in pediatric populations [5]. It is known that familial CCM is autosomal dominant due to mutations in KRIT1 (krev interaction trapped-1), CCM, or programmed cell death 1 [4]. Patients with familial CCM are more likely to develop multiple lesions, and a higher lesion count may lead to an increased chance of intracranial hemorrhage (ICH), seizures, and headaches. Thus, an understanding of a patient’s lesion burden is important in determining patient risk [4].

Familial CCM can be a dynamic condition in which new lesions have been known to form. There is no well-established protocol for routine surveillance of familial CCM so surveillance typically depends on factors like patient preferences and insurance. There is no consensus recommendation for surveillance aside from following clinical judgment [6]. Repeat imaging is indicated when there are changes in symptoms and suspicion of hemorrhage.

This case provides further support that new lesions can develop beyond childhood and into adolescence. This report gives support for possible further research into developing screening and follow-up guidelines for familial CCM given the increased chance of ICH associated with lesion burden and the established possibility for new development of CCM. Due to the established dynamic nature of CCM, follow-up imaging may allow for more accurate risk stratification. Additionally, clinician awareness of possible external factors that can lead to increased risk of new lesions is important for counseling patients. These external factors include cranial radiation, coexistent vascular malformations, hormonal factors, head injury, reactive angiogenesis, and viral infection [7].

Historically, symptomatic lesions have been treated with gamma knife radiosurgery or craniotomy with resection. Seizures are managed with antiepileptic drugs. As advancements continue in the identification of other potential treatments for familial CCM, such as recent trials with propranolol, it is important for patients to be aware of risks so they can make informed decisions regarding treatment and follow-up options [5]. This case reinforces the dynamic nature of familial CCM and the importance of follow-up imaging for lesion burden and risk stratification. 

## Conclusions

This case was put forward to demonstrate the possibility of novel cavernoma development in patients with familial CCM, at least into adolescence. This reinforces the importance of not excluding novel lesions, even in the context of previous normal imaging evaluations. Further research should be pursued regarding the development of more comprehensive protocols for long-term imaging surveillance in familial CCM.
